# *In vivo* multiplex molecular imaging of vascular inflammation using surface-enhanced Raman spectroscopy

**DOI:** 10.7150/thno.28665

**Published:** 2018-11-29

**Authors:** Jonathan Noonan, Steven M. Asiala, Gianluca Grassia, Neil MacRitchie, Kirsten Gracie, Jake Carson, Matthew Moores, Mark Girolami, Angela C. Bradshaw, Tomasz J. Guzik, Gavin R. Meehan, Hannah E. Scales, James M. Brewer, Iain B. McInnes, Naveed Sattar, Karen Faulds, Paul Garside, Duncan Graham, Pasquale Maffia

**Affiliations:** 1 Centre for Immunobiology, Institute of Infection, Immunity and Inflammation, College of Medical, Veterinary and Life Sciences, University of Glasgow, Glasgow, United Kingdom;; 2 Centre for Molecular Nanometrology, Department of Pure and Applied Chemistry, University of Strathclyde, Glasgow, United Kingdom;; 3 Department of Statistics, University of Warwick, Coventry, United Kingdom;; 4 Department of Mathematics, Imperial College London, London, United Kingdom;; 5 The Alan Turing Institute, London, United Kingdom;; 6 Institute of Cardiovascular and Medical Sciences, College of Medical, Veterinary and Life Sciences, University of Glasgow, Glasgow, United Kingdom;; 7 Department of Internal and Agricultural Medicine, Jagiellonian University College of Medicine, Kraków, Poland;; 8 Department of Pharmacy, University of Naples Federico II, Naples, Italy.

**Keywords:** atherosclerosis, molecular imaging, multiplexing, vascular inflammation, surface-enhanced Raman spectroscopy (SERS)

## Abstract

Vascular immune-inflammatory responses play a crucial role in the progression and outcome of atherosclerosis. The ability to assess localized inflammation through detection of specific vascular inflammatory biomarkers would significantly improve cardiovascular risk assessment and management; however, no multi-parameter molecular imaging technologies have been established to date. Here, we report the targeted *in vivo* imaging of multiple vascular biomarkers using antibody-functionalized nanoparticles and surface-enhanced Raman scattering (SERS).

**Methods:** A series of antibody-functionalized gold nanoprobes (BFNP) were designed containing unique Raman signals in order to detect intercellular adhesion molecule 1 (ICAM-1), vascular cell adhesion molecule 1 (VCAM-1) and P-selectin using SERS.

**Results:** SERS and BFNP were utilized to detect, discriminate and quantify ICAM-1, VCAM-1 and P-selectin *in vitro* on human endothelial cells and *ex vivo* in human coronary arteries. Ultimately, non-invasive multiplex imaging of adhesion molecules in a humanized mouse model was demonstrated *in vivo* following intravenous injection of the nanoprobes.

**Conclusion:** This study demonstrates that multiplexed SERS-based molecular imaging can indicate the status of vascular inflammation *in vivo* and gives promise for SERS as a clinical imaging technique for cardiovascular disease in the future.

## Introduction

Atherosclerosis arises from a combination of interacting genetic and lifestyle factors, leading to metabolic dysfunction and chronic inflammation 1,2. It underlies the majority of adverse cardiovascular disease (CVD) outcomes that are the leading cause of death worldwide 3. Diagnostic imaging technologies such as magnetic resonance imaging (MRI), computed tomography (CT), ultrasound (US) and positron emission tomography (PET) have facilitated the anatomic imaging of atherosclerosis; however, these approaches lack the ability to predictively discriminate stable and vulnerable atherosclerotic disease 4-8. Whilst inflammation plays a significant role in atherosclerosis onset and development, as well as plaque vulnerability, this understanding has not yet been successfully translated to allow for disease stratification or the reporting of successful intervention 9-11. In this respect, the inability to image vascular inflammation in patients at the molecular level and identify those with prominent inflammatory disease components has been identified as a key clinical limitation, as this may facilitate cardiovascular risk assessment and management 11,12-14.

Surface-enhanced Raman spectroscopy (SERS) is a novel imaging modality with the potential to directly address these unmet clinical needs. SERS relies on noble metal nanoparticles (NPs) to enhance unique, fingerprint-like Raman signals from reporter molecules at or near the NP surface 15. The spectral diversity of these reporter molecules and amenable NP surface chemistry make SERS highly attractive for multi-parameter molecular imaging 16,17. SERS-active NPs have been successfully engineered to bind to biological molecules, with biofunctional nanoprobes (BFNP) composed of a NP core, reporter molecule, encapsulation layer and targeting biomolecule, facilitating the detection and discrimination of targeted biomarkers 16-19. We previously demonstrated SERS-BFNP imaging of intercellular adhesion molecule (ICAM)-1 in the microvasculature of a murine ear 20; however, whilst it has been shown that multiple BFNPs can be detected in tissues following local or non-targeted administration, targeted multiplex SERS imaging following intravenous injection has yet to be demonstrated 21,22. In addition to ICAM-1, other adhesion molecules such as vascular cell adhesion molecule (VCAM)-1 and P-selectin, contribute to the recruitment of immune cells to atherosclerotic vessels and have been identified as attractive targets for the molecular imaging of vascular inflammation 12,23. A multiplexed approach, reporting all of these markers via SERS-BFNP, could therefore represent a powerful, completely novel semi-quantitative imaging tool, but has yet to be explored in the context of atherosclerosis. Herein, we describe a SERS-BFNP-based strategy that offers the first multiplex targeted *in vivo* imaging of vascular inflammatory biomarkers with scalable potential to contribute across diseases and target tissues.

## Methods

### Mice

NOD *SCID* Gamma mice (NSG) (originally purchased from Jackson Laboratories, Bar Harbor, ME, USA) were bred in-house (Central Research Facility, University of Glasgow, UK) and used for this study. NSG mice were bred in a sterile film isolator and maintained in individually ventilated cages (IVC). Animals were maintained on a 12/12-h light/dark cycle with free access to food and water, and all the procedures were performed in accordance with local ethical and UK Home Office regulations. Animals were randomly assigned to experimental groups and analyzed in a blinded fashion.

### Human adipose engraftment mouse model (^HA^NSG)

Subcutaneous adipose tissue from patients undergoing routine surgery was collected via the Greater Glasgow and Clyde NHS Biorepository. Experiments were approved by the West of Scotland Research Ethics Committee (application number 107, study title: *In situ* nanoparticle assemblies for atherosclerosis diagnosis and therapy). Adipose was dissected from surrounding tissue and cut into segments weighing approximately 0.5 g. Segments were then embedded in Matrigel^TM^ (Corning, Tewksbury, MA) and cultured in endothelial cell culture media MV (Promocell, Heidelberg, Germany) for 7 days to promote the outgrowth of human blood vessels. NSG mice were anaesthetized using isoflurane in the prone position in a laminar flow hood and given Vetergesic (0.1 mg/kg) for pain relief. Mice were shaved using an electric shaver, and the surgery area cleaned using 70% ethanol/chlorhexidine. An incision was made in the back close to the shoulder blades, forceps were inserted and a pouch was created in the subcutaneous space. Matrigel embedded adipose tissue was then inserted into this pouch, and the incision sealed using surgical clips. Mice were allowed recovered on a heat mat within the laminar flow hood and subsequently housed in sterile IVCs. Surgical clips were removed 7 days post-surgery. Engraftment was complete after 21 days. All experiments were carried out between days 21 and 28 days post-surgery.

### Cell culture

Coronary artery endothelial cells (CAEC) (Promocell, Heidelberg, Germany; lot numbers 4071602 and 397Z003) and human umbilical vein endothelial cells (HUVECs) (Thermo Fisher Scientific, Inchinnan, UK; lot number 1578351) were maintained in endothelial cell growth media MV (Promocell, Heidelberg, Germany) supplemented with penicillin/streptomycin at 37 °C/5% CO_2_. 3×10^4^ CAEC/HUVEC were seeded into 8-well slide chambers and rested for 24 h. Cells were then stimulated with 10 ng/mL recombinant Human TNF-α (Peprotech, London, UK) for 24 h.

### Human heart tissue

Human heart tissue collection was performed in collaboration with the Greater Glasgow and Clyde (GG&C) Bio-repository and was approved by the West of Scotland Research Ethics Committee (reference number: 10/S0704/60 and 16/WS/0207; application number 107, study title: *In situ* nanoparticle assemblies for atherosclerosis diagnosis and therapy). Explant human hearts were obtained under informed consent from patients undergoing heart transplantation at the Golden Jubilee National Hospital (GJNH) in Glasgow. Following removal, hearts were immediately placed in cold cardioplegic solution (lactated ringers, 2% St Thomas solution, 5 mEq NaHCO_3_ and 10 mEq KCl; Thermo Fisher Scientific) and 2-3 cm segments of coronary arteries (LAD, RCA or circumflex) were dissected free from the surrounding myocardium before being transferred to the laboratory in cardioplegic solution on ice.

### Processing of coronary arteries for histology, immunofluorescent microscopy and SERS microscopy

Segments of human coronary artery were cryo-embedded in OCT compound (Tissue Tek, Sakura Finetek Europe, Zoeterwoude, the Netherlands). 10 μm thick cryo-sections of tissue were fixed in 10% neutral buffered formalin and subject to hematoxylin and eosin (H&E) staining; lipid distribution in coronary arteries was observed using Oil Red O (ORO) staining. Stained sections were imaged using with an EVOS FL auto microscope (Thermo Fisher Scientific).

### Immunofluorescence microscopy (IFM)

Samples (cells or tissues) were washed twice with PBS and fixed in ice-cold acetone for 5 min at room temperature, air dried, rehydrated in PBS and then blocked for 1 h in Dako Serum-Free Protein Block (DSFPB) (Agilent Technologies, Santa Clara, CA). Samples were incubated with purified primary antibodies (or suitable isotype matched controls) in DSFPB for 2 h at room temperature, washed in PBS, and then probed with Alexa-Fluor 647 labeled anti-Murine IgG raised in goat (Thermo Fisher Scientific) for 30 min. Following washing in PBS, samples were stained with fluorophore-conjugated primary antibodies for 2 h at room temperature if required. Samples were washed in PBS, counterstained with DAPI for 10 min and mounted with a coverslip using Vectashield mounting medium (Vector Laboratories, Burlingame, CA). Immunofluorescence images were acquired using a Zeiss Cell Observer SD confocal fluorescence microscope (Zeiss, Oberkochen, Germany). Fluorochrome-labeled antibodies: AF594 anti-CD31 (catalogue number 303126; BioLegend, San Diego, CA), AF647 anti-mouse IgG raised in goat (catalogue number A21235; Thermo Fisher Scientific). Unconjugated antibodies for IFM: anti-ICAM-1, anti-VCAM-1, anti-P-selectin and IgG1 isotype control (catalogue numbers BBA3, BBA5, BBA30, MAB002 respectively; R&D, Abingdon, UK).

### Transmission electron microscopy (TEM)

CAEC were fixed in 2.5% glutaraldehyde, 4% paraformaldehyde, 0.1 M phosphate buffer, pH 7.2 then washed in 0.1 M phosphate buffer, pH 7.2 and post-fixed in 1% OsO_4_ for 1 h on ice. After several washes in the same buffer, the samples were *en bloc* stained with 0.5% uranyl acetate in water for 30 min. Afterwards, samples were washed with water, dehydrated in ascending acetone series and resin embedded. Ultrathin sections were collected and imaged on a Jeol 1200 Transmission electron microscope (JEOL, Tokyo, Japan). All images obtained were analyzed and processed with FIJI software 24.

### Flow cytometry

Cell viability following exposure to BFNP and comparisons of fresh and implanted adipose tissue were performed using flow cytometry. Cell viability: Confluent HUVEC cultured in 6-well plates were exposed to increasing concentrations of isotype, anti-ICAM-1, anti-VCAM-1, and anti-P-selectin BFNP at a 1:1:1:1 ratio for 24 h. HUVEC were then isolated and suspended in 100 μL PBS containing e780 viability dye (1:1000) (eBioscience, Waltham, MA, USA) and incubated for 10 min at 4 °C. HUVEC were then washed in PBS. Assessment of adipose tissue: The presence of human endothelial cells was determined using flow cytometry. Engrafted mice were culled 21 days post-surgery and their human adipose implants were removed. The tissues were minced and digested in 2% RPMI containing 1 mg/mL collagenase II (Sigma Aldrich, Dorset, UK) and 10 U/mL DNase (Invitrogen, Carslbad, CA, USA) for 50 min at 37 °C under constant agitation. They were then mashed through a 100 µm filter and washed twice in 2% RPMI and once in PBS at 400 ×*g* for 5 min. The cell solutions were then suspended in 400 µL PBS containing e520 viability dye (1:1000) (eBiosciences, Waltham, MA, USA) and incubated for 20 min at 4 °C. The cells were washed in PBS and resuspended in conditioned media from the anti-CD16/CD32 antibody producing hybridoma (2.4G2) (FcBlock). Antibody suspensions containing human Fc Block, V450-labelled anti-human CD36 (both from BD Biosciences, San Jose, USA), APC-e780-labeled anti-human CD45, APC-labeled anti-mouse CD31, PE-labeled anti-human CD31, AF700-labeled anti-human CD34 (all from Biolegend, San Diego, CA, USA), PE-Cy7-labeled anti-mouse CD45 and PerCP-e710-labeled anti-human ICAM-1 (both from eBiosciences, Waltham, MA, USA) were added to each sample and incubated for 20 min at 4 °C. The cells were subsequently washed and analyzed using a BD LSRfortessa flow cytometer (BD Biosciences, San Jose, USA). Analysis of flow cytometry data was performed using FlowJo 10 (FlowJo LLC, Ashland, OR, USA).

### Multiphoton imaging of adipose implants

Multiphoton imaging was performed with a Zeiss LSM7 MP system equipped with 10×/0.3 NA air and a 20×/1.0 NA water immersion objective lenses (Zeiss, Cambridge, UK), a tunable titanium/sapphire solid-state 2-photon excitation source (Chamelon Ultra II; Coherent Inc., Glasgow, UK) and an optical parametric oscillator (OPO; Coherent Inc.). For multiphoton imaging, engrafted mice were given an intravenous injection of 20 µg of PE-labeled anti-human CD31. Following 1 h of incubation, the mice were culled and the implants were removed. Excised adipose tissue was bound with veterinary-grade glue (Vetbond; 3M, MN, US) to a plastic petri dish filled with warmed Ringer's solution. A laser output of 820 nm and OPO signal at 1060 nm provided excitation of adipocytes and hCD31-positive cells. Images were acquired with an X-Y pixel resolution of 512 × 512 in 3 μm Z increments. Images were processed using Volocity 6.1.1 (Perkin Elmer, Cambridge, UK).

### Synthesis of biofunctional gold nanoprobes (BFNP)

Citrate-reduced gold colloid nanoparticles (AuNP) were prepared using a modified Turkevich, Stevenson and Hillier method 25. Briefly, 60.5 mg of sodium tetrachloroaurate dihydrate (Sigma-Aldrich, Dorset, UK) was added to 500 mL dH_2_O and heated until boiling. Upon boiling, an aqueous solution of sodium citrate tribasic dihydrate (Sigma-Aldrich) (57.5 mg in 7.5 mL) was added and boiling was maintained for 15 min. The solution was then allowed to cool to room temperature with continuous stirring maintained throughout. AuNP were functionalized with Raman reporters BPE (Sigma-Aldrich), PPY (Fluorochem, Hadfield, UK), PYOT (Sigma-Aldrich), DP (Sigma-Aldrich) as follows; 990 μL AuNP (52 pM) was mixed with 10 μL of Raman reporter (10 μM) for 30 min at room temperature. The solution was then centrifuged at 2320 ×*g* for 20 min, the supernatant was removed and the pellet resuspended in 1 mL dH_2_O. Antibodies were directly conjugated to polyethylene glycol (PEG) (Thermo Fisher Scientific) as follows: 40 μL of 1 mM polyethylene glycol (PEG; MW 5000) was premixed with 74 μL of 1 mg/mL N-(3-dimethylaminopropyl)-N′-ethylcarbodiimide (Sigma-Aldrich). Twenty μL of the desired antibody (0.5 mg/mL anti-ICAM-1, anti-VCAM-1, anti-P-selectin or murine IgG1 isotype control; catalogue numbers BBA3, BBA5, BBA30, MAB002 respectively; R&D, Abingdon, UK) was premixed with 217 μL of 1 mg/mL N-hydroxysulfosuccinimide sodium salt (Sigma-Aldrich). The two premixed solutions were combined, 709 μL of 10 mM HEPES (pH 7) buffer (Sigma-Aldrich) was added, and then the solution was gently agitated overnight. BFNP were completed by combining Raman reporter-functionalized AuNP with the antibody/PEG solution for 2-3 h. BFNP were then centrifuged at 2320 ×*g* for 20 min, the supernatant was removed, and the pellet was resuspended in 100 μL bovine serum albumin/sodium azide solution (0.1% / 2 mM; Sigma-Aldrich). Assembled conjugates typically had diameters around 80 nm, UV-VIS lambda max near 530 nm (data not shown), and zeta potentials below -25 mW. Further details on the spectra and chemical structure of the Raman reporters used in this study can be found in 26.

### Biofunctional nanoprobe (BFNP) characterisation

BFNP were characterized for size, stability and SERS signal using UV-Visible spectroscopy, dynamic light scattering (DLS) and surface-enhanced Raman spectroscopy (SERS). UV-vis spectroscopy was carried out on a Varian Cary 3000 BioUV-Visible spectrophotometer (Agilent Technologies, CA, USA) with Win UV scan application version 2.00 software. The scanning wavelength range was 300-800 nm. All measurements (1 mL) were performed in disposable PMMA microcuvettes. Size and zeta measurements for each sample were recorded using a Malvern Zetasizer Nano ZS (Malvern, Worcestershire, UK) along with Zetasizer µV and APS version 6.20 software. All samples (1 mL) were run in disposable PMMA macrocuvettes with a standard Malvern Dip Cell used for zeta measurements. SERS analysis of each sample was performed using the Snowy Range Sierra Series (Snowy Range Instruments, WY, USA) with an excitation wavelength of 638 nm. Samples were analyzed using clear glass vials (Sigma Aldrich, Dorset, UK). Instrument settings for each sample were 40 mW laser power with a 3 s acquisition time.

### *In vitro* immuno-SERS staining for SERS microscopy

CAEC/HUVEC were cultured in 8-well EZ chamber slides (Merck Millipore, Burlington, MS) in unstimulated or TNF-α-stimulated conditions. Fixed cells: Cells were briefly fixed in ice-cold acetone then exposed to BFNP in bovine serum albumin/sodium azide /NaN_3_ solution at room temperature for 2 h. Cells were then washed thoroughly with PBS, rinsed in distilled water, and allowed to air dry for imaging. Live cells: CAEC were exposed to BFNP in endothelial cell growth media MV (Promocell, Heidelberg, Germany) at 37 °C/5% CO_2_ for up to 24 h. Cells were then washed thoroughly with PBS and fixed in 4% paraformaldehyde for 10 min. Slides were rinsed in distilled water and allowed to air dry prior to imaging.

### BFNP targeting of human coronary artery segments

Coronary arteries were sealed with ligatures at each end prior to injection with BFNP resuspended in endothelial cell growth media MV (Promocell). Vessels were then incubated with BFNP for 12 h at 37 °C/5% CO_2_. Sutures were then removed and unbound BFNP were removed with multiple PBS washes. Vessels were then subjected to 785 nm SERS spectroscopy, subsequently cryo-embedded in OCT compound and cut into 10 μm sections using a cryostat (Thermo Fisher Scientific) to facilitate analysis by SERS microscopy or IFM.

### BFNP targeting *in vivo*

^HA^NSG mice were injected with BFNP intravenously. SERS spectroscopy was then used to investigate grafts, liver, or small blood samples for SERS signal. Alternatively, or in addition, grafts were excised, cryo-embedded in OCT compound, cut into 10 μm sections using a cryostat, and subjected to SERS/IFM microscopy.

### SERS microscopy

Raman and darkfield images were acquired on a Renishaw InVia Raman microscope running WiRE 4.3 software (Renishaw, Wotton-under-Edge, UK). The system was configured to utilize an upright microscope, piezo stage, 633 nm (HeNe) excitation, 50×/0.75 NA Leica darkfield objective (Leica Microsystems, Cambridge, UK), 1800 l/mm grating and either the native Renishaw CCD camera or Andor EMCCD. Experimental parameters, including collection time (0.2 s Renishaw, 0.075 s EMCCD) and laser power (0.5 mW at sample) were optimized to ensure optimal SERS signal was observed from BFNP. The size of individual Raman maps varied from scan to scan; however, the same step size (1 μm) was used in both the x and y directions for each scan. Scan areas are highlighted throughout by a black box with a broken line.

### Processing of SERS microscopy images

Cosmic ray removal, background subtraction, spectral smoothing (n=5 pixels) and direct classical least squares (DCLS) analysis were performed in the WiRE 4.3 software. False color images were generated from the DCLS results, with the look-up table thresholds set to minimize the influence of noise and highlight the Raman spectra in the map in greatest agreement with reference BFNP spectra. Look-up table settings for all Raman images are shown in **Figure S1**.

### SERS spectroscopy

Raman spectra from coronary arteries and animals treated with BFNP were collected using an in-house instrument described previously 27. Briefly, the system consists of a attenuable 785 nm laser (Innovative Photonics Solutions, Monmouth Junction, NJ), a single fiber optic Raman probe with internal filtering (Wasatch Photonics, Durham, NC) mounted on an *xyz* stage (ThorLabs, Newton, NJ) and a fixed-grating spectrometer with thermoelectric cooling (WP 785, Wasatch Photonics). This excitation wavelength was selected for spectroscopy due to lower tissue absorption and damage when analyzing tissues *ex*/*in vivo* whilst maintaining signal detection efficiency. Typical spectral acquisition settings consisted of 1 s acquisitions with 30-40 mW of power at the sample, depending on the prominence of auto-fluorescence background. Background contributions were removed in WiRE 4.3, as in microscopy images.

### Quantification of SERS spectra using DCLS

Direct classical least squares data analysis (DCLS) was performed in the Wire 4.3 software. Prior to DCLS fitting, cosmic ray removal, background subtraction, and spectral smoothing (n=5 pixels) were performed on individual spectra from each Raman map. All spectra in the map were then averaged, creating a single representative spectrum per 2D Raman scan. DCLS was performed on these spectra using four reference spectra, one from each of the three targeted particles and the isotype control, resulting in a score for each Raman reporter for each averaged spectrum. Each Raman reporter score was averaged from 12 imaged cells per condition, with the standard deviation representing the error.

### Quantification of SERS spectra using Bayesian modeling

Bayesian statistical analysis was performed in the R statistical computing platform 28. Prior to Bayesian fitting, cosmic ray removal was conducted on individual spectra from each Raman map. All spectra in each map were then averaged, creating a single representative spectrum per 2D Raman scan. Bayesian linear regression was then performed using four reference spectra and a penalized cubic spline modeling the baseline as in Moores et al. 29. For baseline modeling, baseline knots were placed 5 wavenumbers apart. For the prior distributions of the scores, truncated normal distributions with mean 0, standard deviation 4000 and truncation at 0 were selected as scores must be non-negative. For the baseline parameters, we chose a multivariate Normal distribution with mean 0, and precision matrix 10^-6^ (I + λF^T^F), where I is the identity matrix, λ is the roughness penalty, and F is the matrix of second order finite difference coefficients. For each representative spectrum, λ was chosen by fitting a penalized cubic spline to a region with no peaks. Finally, for the variance of the errors, we chose an inverse-Gamma distribution with shape 0.001 and scale 2500. We obtained 1000 sequential Monte Carlo samples from the relevant posterior distribution for each spectrum 29. Spectra were then grouped according to stimulation condition and 12 posterior sample members were averaged within each group to give 1000 average scores per reference spectra per stimulation condition.

### Statistical analysis

Statistical analyses were performed using GraphPad Prism Version 6.0 (GraphPad, CA, USA). Statistical significance was calculated using ordinary one-way ANOVA with *post-hoc* Tukey's test, and R^2^ values were calculated using linear regression. Statistical significance was defined as *P*<0.05. We used Bayesian linear regression 30 to obtain posterior probabilities of differences between means from the 1000 average scores. In this case, significance was defined as <5% overlap between the scores.

## Results

### Synthesis of SERS-active BFNP for SERS molecular imaging

BFNP were designed and synthesized consisting of a surface-enhancing gold core, a Raman reporter to endow a unique Raman spectral signature, polyethylene glycol (PEG) for stability, biocompatibility and prolonged *in vivo* circulation times, and antibodies to provide target molecule specificity. A BFNP schematic is shown in **Figure S2A**. The ability to discriminate multiple BFNP with different molecular targets was achieved by pairing each antibody/NP combination with a unique Raman reporter. The spectrum of each Raman reporter utilized is shown, with the unique spectral features used for their identification highlighted (**Figure S2B**). The primary antibody/reporter combinations selected were: isotype-DP (4,4- dipyridyl), identified by a peak at 1296 cm^-1^; anti-ICAM-1-BPE (1,2-bis(4-pyridyl)ethylene), identified by a peak at 1202 cm^-1^ (in a multiplex, this shift appears as a deviation or “shoulder” on a larger peak at 1215 cm^-1^); anti-VCAM-1-PYOT(5-(pyridine-4-yl)-1,3,4-oxadiazole-2-thiol), identified by a peak at 1575 cm^-1^; anti-P-selectin-PPY (4-(1H-pyrazol-4-yl)pyridine), identified by a peak at 952 cm^-1^. A Raman shift common to all BFNP was observed at 1605 cm^-1^ and was used to indicate the general presence of BFNP in the absence of delineating spectral features. BFNP were ~80 nm in diameter, regardless of the specific functionalization (**Figure S2C**), with Zeta potentials below -25 mV demonstrating chemical stability 31 (**Figure S2D**). BFNP displayed no evidence of toxicity at 20 μg/L, the maximum concentration used on live cells *in vitro* (**Figure S3**).

### SERS imaging of BFNP facilitates simultaneous and quantifiable multiplexed detection of adhesion molecules on human endothelial cells *in vitro*

#### SERS-BFNP facilitates adhesion molecule imaging in fixed endothelial cells

**Figure 1:**
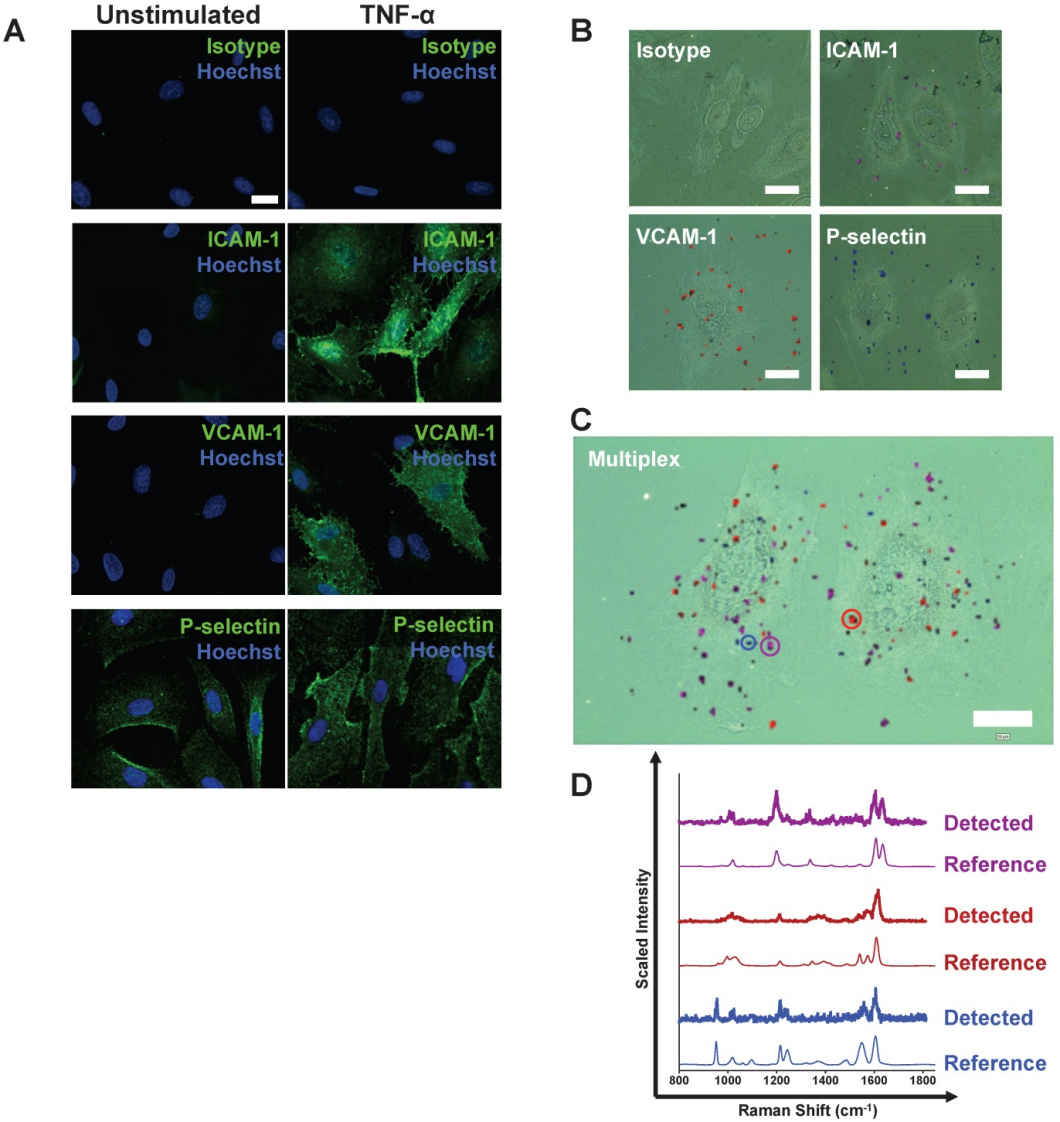
** Following stimulation, coronary artery endothelial cells (CAEC) express adhesion molecules detectable via immuno-SERS imaging in single and multiplex formats. (A)** Fluorescence images of immunohistochemical staining of ICAM-1, VCAM-1 and P-selectin on CAEC in unstimulated and 10 ng/mL TNF-α-stimulated conditions. Isotype control, ICAM-1, VCAM-1 and P-selectin staining shown in green; nuclei were counterstained using Hoechst 33342 (blue). **(B)** CAEC were stimulated with 10 ng/mL TNF-α for 24 h, fixed in acetone, and incubated with isotype control, anti-ICAM-1, anti-VCAM-1 or anti-P-selectin BFNP or **(C)** with all BFNP simultaneously before being subjected to SERS mapping. **(D)** Representative spectra from anti-ICAM-1 (purple), anti-VCAM-1 (red) and anti-P-selectin (blue) BFNP acquired from the color-matched circles in (C) are shown above their respective reference spectra. Optical images in (B-C) are darkfield images. Scale bars = 20 μm. Results are representative of 3 independent experiments.

An immunohistochemistry (IHC)-like approach in tumor necrosis factor (TNF)-α-stimulated human coronary artery endothelial cells (CAEC) was used initially. Stimulated cells expressed ICAM-1, VCAM-1 and P-selectin as demonstrated by conventional immunofluorescence (**Figure 1A**)*.* The ability of anti-ICAM-1-BPE, anti-VCAM-1-PYOT and anti-P-selectin-PPY BFNP to bind to their respective targets individually on acetone-fixed endothelial cells was investigated (**Figure 1B**). SERS microscopy analysis of TNF-α-stimulated CAEC identified the binding of each targeted probe to its specific target in isolation, but, as expected, the DP-labeled isotype control probes did not bind. We confirmed that the choice of reporter molecules did not influence binding of BFNP to target molecules by showing that anti-ICAM-1 BFNP functionalized with PPY, BPE, PYOT and DP produced similar results, whilst isotype equivalents produced low-to-no signal (**Figure S4**). The simultaneous detection and discrimination of ICAM-1, VCAM-1 and P-selectin was demonstrated by exposing activated CAEC to an equimolar mixture of anti-ICAM-1-BPE, anti-VCAM-1-PYOT, anti-P-selectin-PPY and isotype-DP probes, with isotype probes producing low-to-no signal in all experiments (**Figure 1C**). Darkfield images indicated the presence of BFNP on cells, appearing as small bright spots due to their unique scattering properties, whilst delineation of particle type was achievable via the Raman spectra collected. Examples of individual spectra acquired from within the Raman maps are shown, confirming that the experimentally observed spectra from specific points in the Raman map match prototypical reference spectra for each of the three targeted probes (**Figure 1D**). These results demonstrate the multiplexed detection of three adhesion molecules using our SERS-BFNP approach in fixed endothelial cells.

#### SERS-BFNP approach facilitates adhesion molecule imaging in live endothelial cells

SERS-BFNP imaging in live endothelial cells was shown to be successful by exposing TNF-α-stimulated CAEC to a mixture of anti-ICAM-1-BPE, anti-VCAM-1-PYOT, anti-P-selectin-PPY and isotype-DP probes (**Figure S5A**). Using Raman and darkfield imaging, an increase in the amount of anti-ICAM-1 BFNP was observed when incubation with the probe mixture was increased from 30 min to 3 h; this was not observed with anti-VCAM-1 or anti-P-selectin probes. We hypothesize that the increase in anti-ICAM-1 probe signal may be as a result of BFNP uptake by CAECs. Transmission electron microscopy (TEM) analysis of TNF-α-stimulated CAECs exposed to single varieties of BFNP, to facilitate differentiation of particles in the absence of correlated spectral information afforded by SERS, identified an abundance of anti-ICAM-1 NPs located within cytoplasmic vesicles after 24 h. Anti-VCAM-1, anti-P-selectin and isotype BFNP were detected infrequently or not at all (**Figure S5B**). These data confirm the ability of BFNP to bind to their molecular targets and be detected using SERS microscopy in live cells, whilst highlighting that ICAM-1 targeted probes can be internalized by CAECs.

#### Adhesion molecule expression is semi-quantifiable using SERS-BFNP approach

**Figure 2:**
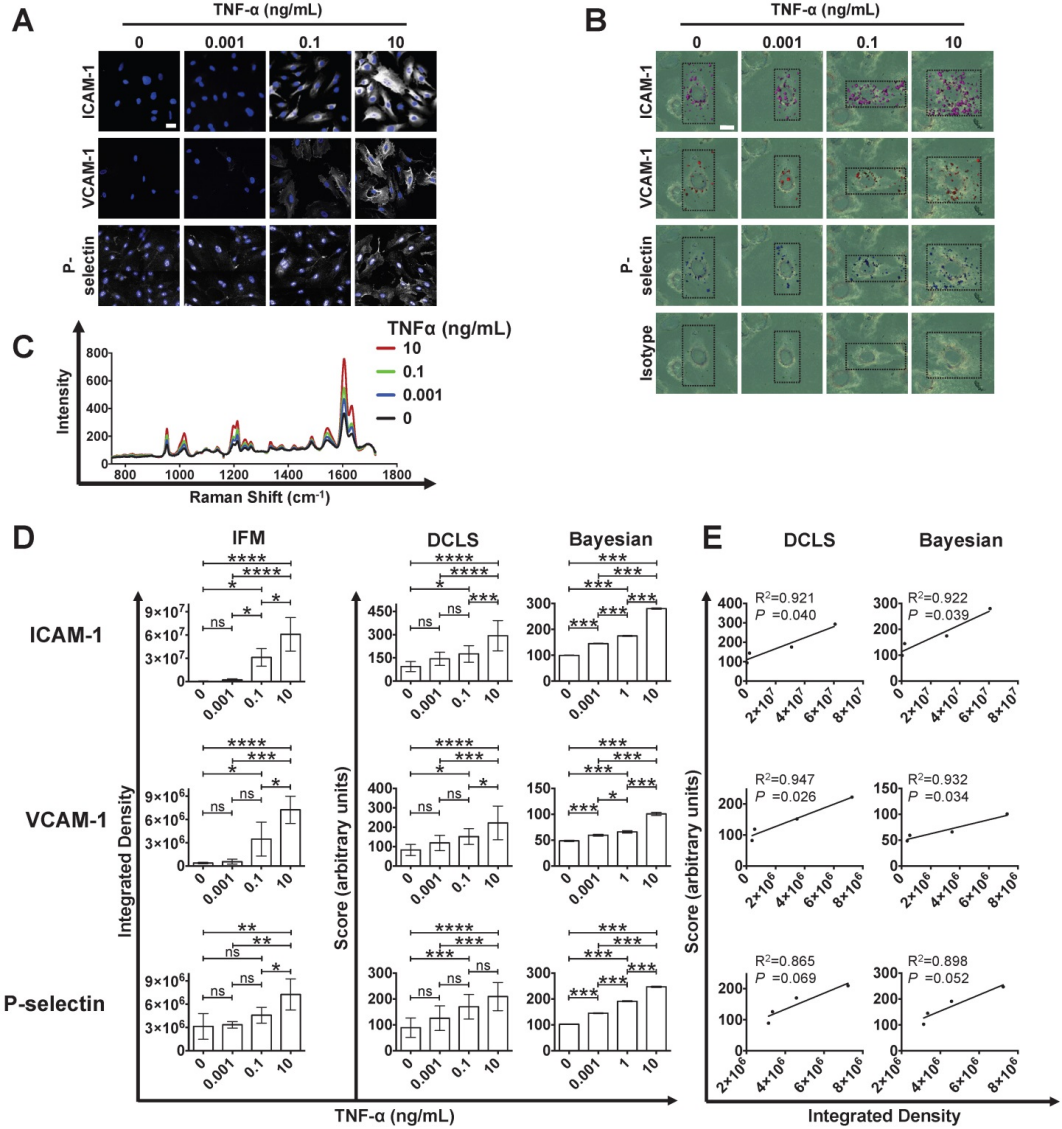
** Quantification of adhesion molecule expression by SERS-BFNP molecular imaging using DCLS and Bayesian methodologies. (A)** Immunofluorescence staining of ICAM-1, VCAM-1 and P-selectin on human umbilical vein endothelial cells cultured for 6 h in unstimulated, 0.001, 0.1 or 10 ng/mL TNF-α-stimulated conditions. Adhesion molecule staining is shown in white; all cells were counterstained with Hoechst 33342 to identify nuclei (blue). **(B)** HUVEC were cultured unstimulated or stimulated with 0.001, 0.1 or 10 ng/mL TNF-α for 6 h, fixed in acetone, and incubated with anti-ICAM-1, anti-VCAM-1, anti-P-selectin, and isotype control BFNP simultaneously. Cells were then subjected to SERS mapping. Each channel from representative multiplex images are shown for: anti-ICAM-1 (purple), anti-VCAM-1 (red), anti-P-selectin (blue), isotype (green) BFNP. **(C)** For quantification, spectra from each SERS microscopy image were averaged to provide one spectrum per image; a representative spectrum for each TNF-α condition is shown. **(D)** Integrated densities alongside DCLS and Bayesian SERS quantification scores were calculated for ICAM-1, VCAM-1 and P-selectin expression from each TNF-α-stimulated condition. **(E)** The correlation between immunofluorescence integrated density and DCLS/Bayesian quantification methodologies and their respective R^2^ values are shown. Optical images in (B) are darkfield images. Scale bars = 20 μm. Each Raman reporter score was averaged from 12 imaged cells per condition. Values are mean ± SD. *P<0.05; **P<0.01; ***P<0.001; ****P<0.0001. In the Bayesian quantification, we indicate where the posterior probability of the difference between the group means being greater than zero is >95% (*), >99% (**), >99.9% (***).

Information obtained from Raman imaging of the BFNP on fixed cells was used to develop a semi-quantitative approach to monitor changes in biomarker expressions. Human umbilical vein endothelial cells (HUVEC) were employed, which demonstrated a lower basal but similar induced expression of adhesion molecules in comparison with CAECs (data not shown). Conventional immunofluorescence microscopy (IFM) analysis of HUVEC identified a concentration-dependent response to TNF-α in respect to adhesion molecule expression (**Figure 2A**). In parallel, stimulation of HUVEC with increasing TNFα concentrations increased binding of anti-ICAM-1-BPE, anti-VCAM-1-PYOT and anti-P-selectin-PPY, but not isotype-DP BFNP, as measured by multiplexed SERS microscopy (**Figure 2B**). In order to quantify these results, the cumulative spectra of each Raman/SERS map were averaged to establish one representative spectrum per SERS microscopy image. The increasing adhesion molecule SERS signal observed by Raman/SERS mapping was conserved in these spectra (**Figure 2C**), with the spectral intensity at Raman shifts related to each of the BFNP increasing with TNF-α concentration.

Quantification of immunofluorescence images confirmed the concentration-response relationship between TNF-α stimulation and HUVEC expression of ICAM-1, VCAM-1 and P-selectin (Figure 2D). This single marker immunofluorescence quantification was as a standard with which to compare two multiplexed SERS quantification methodologies: one using direct classical least squares (DCLS) and the other using a Bayesian approach. Two quantification methods were explored as a standard method of quantification that is rigorous and robust enough for broad application; a unanimous model has yet to be defined due to the novelty of SERS quantification methodologies. Crucially, the quantification scores for both DCLS (**Figure S6A**) and Bayesian (**Figure S6B**) approaches increased linearly with BFNP concentration, indicating their suitability as a means of quantifying SERS signals. Both quantification methodologies produced near-zero scores in all stimulation conditions explored for isotype control BFNP (**Figure S7**). DCLS analysis of stimulated HUVEC with multiplexed BFNP produced a similar concentration-response relationship as observed with single marker IFM, with different stimulation conditions resulting in observation of significant differences for each adhesion molecule (**Figure 2D**). Additionally, by using a Bayesian approach, which quantifies each spectrum 1000 times, a TNF-α concentration response was found that was similar to that observed with IFM, further corroborating the DCLS analysis (**Figure 2D**). IFM analysis correlated highly with both DCLS and Bayesian SERS quantification methodologies (**Figure 2E**). In summary, SERS imaging of BFNP can be used for the quantifiable multiplex detection of adhesion molecules on human endothelial cells *in vitro*.

### SERS imaging of BFNP facilitates simultaneous multiplex detection of adhesion molecules in human coronary artery *ex vivo*

**Figure 3:**
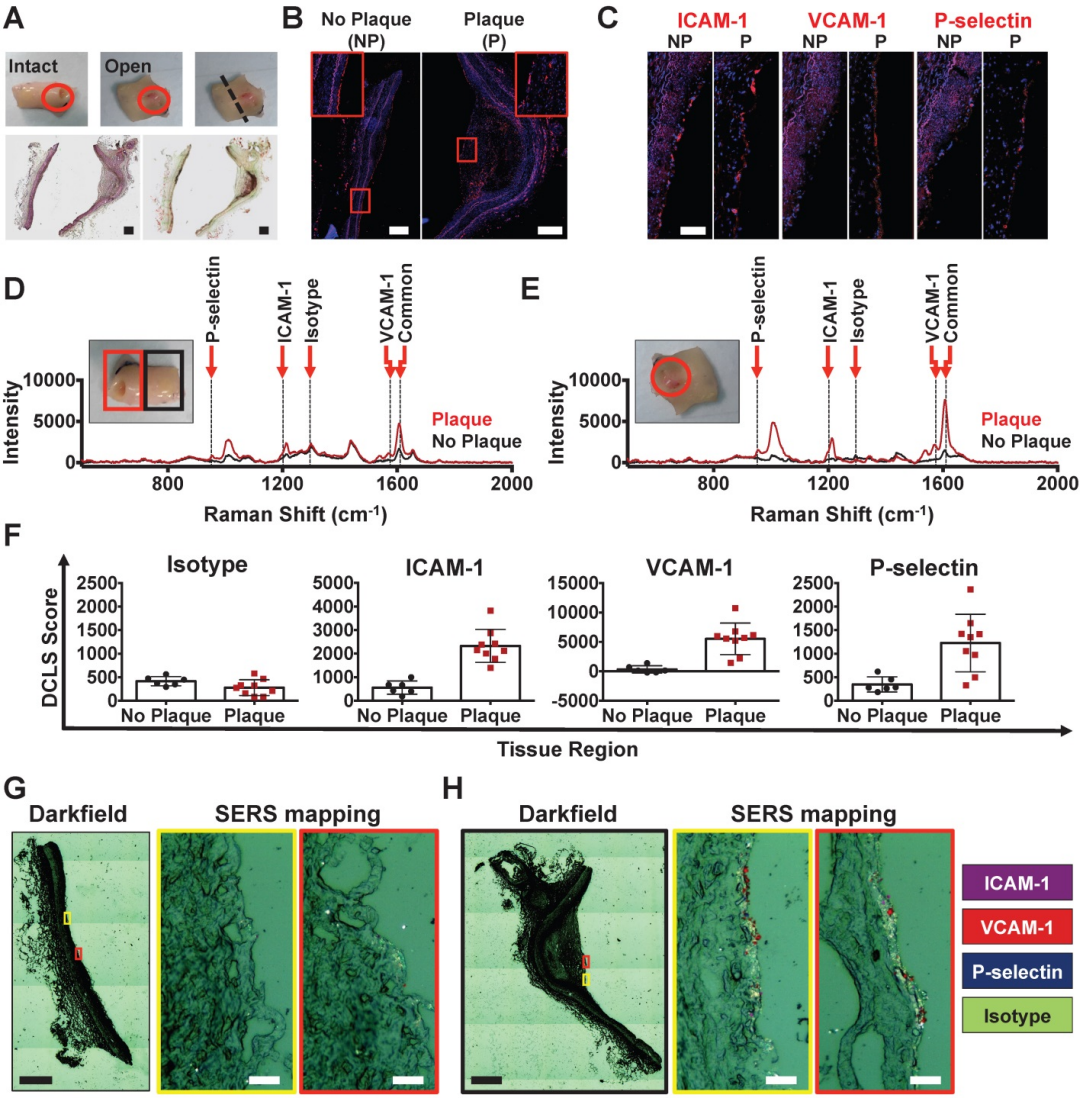
** SERS-BFNP molecular imaging of atherosclerotic coronary arteries.** A single human coronary artery was isolated from the heart of a patient undergoing heart transplantation surgery. The lumen of the artery segment was then injected with a mixture of anti-ICAM-1, anti-VCAM-1, anti-P-selectin, and isotype control BFNP, sutured closed, and incubated at 37 °C/5% CO_2_ for 12 h. Sutures were then removed and the artery segment was thoroughly washed prior to SERS spectroscopy and subsequent analysis of morphology, expression of adhesion molecules and SERS mapping. **(A)** Photos are shown of an intact (upper left panel) and dissected *en face* opened (upper middle panel) atherosclerotic artery. The plaque location is highlighted with a red circle. Following SERS spectroscopy analysis, the opened artery was bisected to separate atherosclerotic and non-atherosclerotic regions of the vessel; the cut location is highlighted with a black dashed line (upper right panel). H&E (lower left panel) and Oil Red O staining (lower right panel) were carried out to investigate vessel morphology and lipid deposits, respectively. **(B)** Immunofluorescence staining for CD31, and expression of **(C)** ICAM-1, VCAM-1 and P-selectin are shown in red. Nuclei were counterstained using Hoechst 33342 (blue). **(D-E)** SERS spectroscopy was conducted on atherosclerotic (red lines) and non-atherosclerotic (black lines) regions of the intact (D) and opened (E) artery. The unique peaks and common peak used to identify each BFNP configuration are highlighted with a red arrow and dotted line, and labeled accordingly. Spectroscopy results are displayed as spectra averaged from at least 3 different points within the atherosclerotic and non-atherosclerotic regions. **(F)** ICAM-1, VCAM-1, P-selectin, and isotype signals were quantified using DCLS, with each point representing a spectrum acquired from a different location with either atherosclerotic or non-atherosclerotic regions of the opened vessel. **(G-H)** SERS mapping was then carried out for anti-ICAM-1 (purple), anti-VCAM-1 (red), anti-P-selectin (blue), and isotype (green) BFNP. Darkfield images are shown and the regions of non-atherosclerotic (G) and atherosclerotic (H) artery subject to SERS mapping are highlighted with yellow and red boxes corresponding to SERS maps on the right of each darkfield image. Optical images in (G-H) are darkfield images. Scale bars: (A-B) = 500 μm; (C) = 100 μm; (G-H) = 500 μm (black bar) and 20 μm (white bar).

The SERS detection approach was applied in human tissue *ex vivo* using a single Raman fiber probe-configured, custom-made spectroscopic system 27. Fresh segments of human coronary arteries from patients undergoing heart transplantation were obtained for this study. Clinical characteristics and summary SERS-BFNP results for all vessels are shown in **Table S1**. Vessels which did not present any evidence of atherosclerosis did not lead to the detection of significant adhesion molecules using any spectroscopic approach (**Figure S8**). The artery shown in **Figure 3** contained an atherosclerotic plaque, with tissue remodeling and lipid accumulation evident (**Figure 3A**), that also had an intact endothelium (**Figure 3B**). ICAM-1 and P-selectin were present on both atherosclerotic and non-atherosclerotic regions of tissue, whilst VCAM-1 was only identified on atherosclerotic tissue (**Figure 3C**).

Exposure of the artery to an equimolar mixture of anti-ICAM-1-BPE, anti-VCAM-1-PYOT, anti-P-selectin-PPY and isotype-DP BFNP facilitated SERS spectroscopic detection of ICAM-1, VCAM-1 and P-selectin on the plaque, with higher ICAM-1 and P-selectin intensity than in non-atherosclerotic regions, and VCAM-1 found only on the atherosclerotic region (**Figure 3D**). To confirm the accuracy of these results, the artery was opened and the Raman spectroscopic investigation was repeated, allowing more rigorous control of the location being analyzed and improved sampling (**Figure 3E**). This analysis confirmed the detection of ICAM-1, VCAM-1 and P-selectin on atherosclerotic tissue. Using the DCLS quantification strategy, the results obtained from this artery were quantified, producing a numerical trend towards increased adhesion molecule expression, but no change in isotype signal, when comparing atherosclerotic to non-atherosclerotic regions of tissue (**Figure 3F**).

Both the atherosclerotic and non-atherosclerotic regions of tissue were subsequently examined by SERS microscopy, which confirmed the high positivity of ICAM-1, VCAM-1 and P-selectin on atherosclerotic endothelium, and only infrequent detection of ICAM-1 and VCAM-1 on non-atherosclerotic tissue (**Figure 3G**-**H**). Furthermore, BFNP-related darkfield contrast at the endothelial surface of atherosclerotic tissue was visibly increased compared with that at non-atherosclerotic regions, suggesting far greater BFNP density at this tissue site. In summary, these data confirm the suitability of this SERS-BFNP platform for simultaneous multiplexed detection of adhesion molecules in human atherosclerotic artery *ex vivo*.

### SERS imaging of BFNP facilitates simultaneous multiplex detection of adhesion molecules *in vivo*

**Figure 4:**
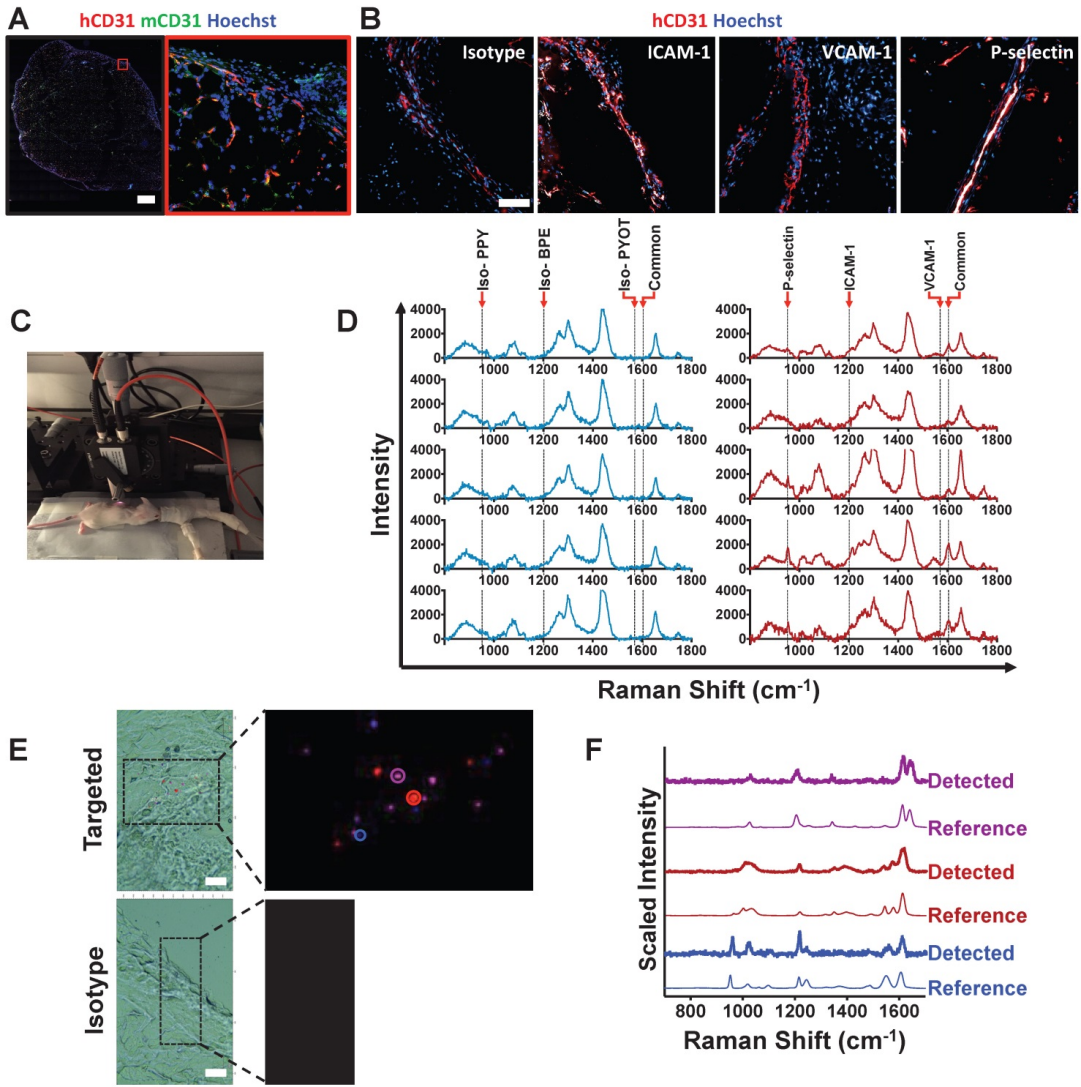
***In vivo* SERS-BFNP molecular imaging of adhesion molecules.** Following engraftment of human adipose, ^HA^NSG mice were allowed to recover for 3 weeks. Mice were then injected intravenously with 5 μg of human recombinant TNF-α 4 h prior to receiving an intravenous injection of BFNP. **(A)** Following SERS-BFNP molecular imaging, adipose grafts were excised and immunofluorescently stained for human (red) and murine (green) CD31.** (B)** Isotype control, ICAM-1, VCAM-1, and P-selectin staining are also shown in white counterstained with human CD31 (red). Nuclei were counterstained using Hoechst 33342 (blue). **(C)** To conduct SERS-BFNP molecular imaging, ^HA^NSG mice were anaesthetized and their adipose grafts non-invasively analyzed *in vivo* using SERS spectroscopy. **(D)** SERS spectra were acquired from mice that received a mixture of isotype-PPY, -BPE and -PYOT (blue spectra), or anti-P-selectin-PPY, anti-ICAM-1-BPE, anti-VCAM-1-PYOT BFNP (red spectra). The spectra shown are from 5 isotype vs. 5 targeted mice, with each spectrum acquired from a different mouse. **(E)** In addition to immunofluorescence microscopy, excised adipose grafts were analyzed using SERS microscopy. Detection of BFNP from sections of adipose tissue isolated from ^HA^NSG mice that received anti-ICAM-1 (purple), anti-VCAM-1 (red), and anti-P-selectin (blue) (upper panels) or Isotype-BPE (purple), Isotype-PYOT (red), and Isotype-PPY (blue) (lower panels) are shown superimposed on darkfield tissue images alongside a magnified image of Raman maps from the scanned areas (black boxes). The colored circles in the Raman map ((E) upper panel) correlate to the acquired spectra shown in **(F)** above their respective reference spectra. The optical image in (E) is a darkfield image. Scale bars: (A) = 1000 μm; (B) = 100 μm; (E) = 20 μm.

The ultimate test is whether the SERS-BFNP approach could be applied for molecular imaging of adhesion molecules *in vivo*. To achieve this, a humanized mouse model (^HA^NSG model) was used whereby NSG mice, which are amenable to xenografting due to their immunocompromised status 32,33, were engrafted with human subcutaneous adipose tissue. Adipose engraftment resulted in NSG mice containing viable and perfused human microvasculature (**Figure S9**). The presence of human vessels, which were identified microscopically in close proximity to the murine vasculature, was confirmed (**Figure 4A**). Furthermore, it was found that following an intravenous injection of TNF-α, these vessels expressed ICAM-1 and P-selectin; in contrast, there was no evidence of VCAM-1 (**Figure 4B**). To test SERS-BFNP molecular imaging *in vivo*, ^HA^NSG mice were intravenously injected with TNF-α, and 4 h later 34 received BFNP functionalized with anti-ICAM-1-BPE, anti-VCAM-1-PYOT and anti-P-selectin-PPY; control mice received isotype-BPE, isotype-PYOT and isotype-PPY. These mice were then subjected to non-invasive *in vivo* SERS spectroscopy, as demonstrated in **Figure 4C**. At 24 h post injection, there was little to no signal from BFNP in the blood, suggesting clearance from the circulation, with BFNP signal from the liver being similar for both isotype and targeted probes at 1 h and 24 h post-BFNP injection (**Figure S10**).

SERS spectra at 24 h following BFNP injection in mice that received a mixture of targeted BFNP demonstrated ICAM-1 and P-selectin single detection, as well as their simultaneous duplex detection. The peak common to all reporter molecules is present in all spectra from the targeted mice; in contrast, BFNP signals were not observed in mice receiving equivalent isotype controls. Spectra obtained from 5 isotype and 5 targeted mice are shown (**Figure 4D**). Subsequent SERS microscopy analysis identified the presence of ICAM-1, VCAM-1 and P-selectin in mice that received targeted BFNP, but showed no evidence of isotype probes in the control group (**Figure 4E**). Examples of individual SERS spectra acquired within microscopy images are shown, confirming that the experimentally observed spectra from specific points in the Raman image match prototypical reference spectra for each of the three targeted probes (**Figure 4F**). In summary, these data demonstrate the successful application of a SERS-BFNP imaging approach *in vivo* for multiplexed non-invasive imaging of adhesion molecules.

## Discussion

In this study, we have demonstrated the feasibility of a SERS-BFNP molecular imaging platform for non-invasive, simultaneous targeted multiplexed detection of adhesion molecules *in vivo* in the context of vascular inflammation. There is great interest in the ability to image specific inflammatory biomarkers from the clinical perspective, as inflammation plays fundamental roles in atherosclerosis, but at present there are significant limitations in the potential to observe these molecules in patients. As such, this study will have important implications for potentiating the development of clinical SERS-BFNP molecular imaging systems in the cardiovascular setting and in any other pathology in need of multiplexed biomarker imaging.

Molecule-specific BFNP were developed—a strategy employed in several other studies 16,17,19-22,35—as a means of detecting adhesion molecules using SERS. We have targeted adhesion molecules as a proof of concept, given that they are easily accessible to intravenously injected contrast agents. The capability of the BFNPs to bind specifically to their molecular targets, facilitating simultaneous imaging of ICAM-1, VCAM-1 and P-selectin, has been convincingly demonstrated using SERS microscopy. Supporting previous studies using BFNP as drug delivery systems 36,37, it was found that ICAM-1-targeted particles were internalized by CAECs, resulting in amplification of the SERS signal and presenting a mechanism that could be manipulated for theranostic drug delivery or improving imaging depth.

Quantification of adhesion molecule expression using the SERS-BFNP molecular imaging system was achieved and, in the process, demonstrated that both DCLS and Bayesian multiplexed quantification approaches correlated well with a classical immunofluorescence methodology *in vitro*. Building on the work of the Liu group 38-43, who have developed a semi-quantitative SERS-BFNP approach for tumor phenotyping, an isotype BFNP was included in the multiplex probe panel as an internal standard for non-specific binding. Due to variable levels of non-specific binding observed in different tumor types, the phenotyping of tumors in the referenced studies required ratiometric normalization of biomarker scores to isotype BFNP scores. In comparison, normalization was not required in this study due to near zero scores for isotype-BFNP in all conditions analyzed. The ability to quantify vascular inflammatory biomarkers would be most advantageous, as it is the balance between pro- and anti-inflammatory mechanisms that will define pathological outcomes 2,44,45.

Raman spectroscopy imaging approaches, which focus on detecting the native spectral fingerprints of tissue components such as calcium and collagen, have demonstrated the ability to differentiate atherosclerotic and non-atherosclerotic arteries with reasonable success 46-48. However, acquisition of Raman signals from atherosclerotic plaques *in vivo* requires the use of invasive intravascular probes due to the inherent lack of tissue penetration commonly associated with Raman spectroscopy. By comparison, the use of targeted BFNP in a SERS approach greatly expands the number of biomarkers one could image, whilst providing the sensitivity needed to detect spectra through several centimeters of tissue, thus offering the potential for non-invasive use in superficial arteries 27,49,50. To put the multiplexing potential into context, identification and discrimination of 10 non-targeted SERS nanoprobes has been demonstrated *in vivo*, making expansion of the number of target molecules simultaneously imaged a distinct possibility in the future 51.

The SERS-BFNP molecular imaging approach used here successfully detected adhesion molecules in fresh human coronary artery. Specifically, SERS spectroscopic detection of adhesion molecules was only achieved in atherosclerotic arteries, highlighting the potential for discriminating atherosclerotic from non-atherosclerotic tissue. The incorporation of additional biomarkers related to inflammation, angiogenesis or thrombotic pathways would allow increased sensitivity of detection and potentially plaque phenotyping in respect to detecting early stage lesions and atherosclerosis at high risk of causing a clinically significant event. Furthermore, the DCLS quantification approach tested *in vitro* verified our interpretations of plaque vs. non-plaque spectra in a human coronary artery, with increased ICAM-1, VCAM-1 and P-selectin signal in the atherosclerotic tissue regions. Though there are a low number of replicates due to the limited availability of these valuable tissues, these results demonstrate SERS-BFNP multiplex molecular imaging of adhesion molecules in human coronary arteries, whilst the data also suggests that DCLS quantification may be applicable to spectra obtained from tissues.

The limited number of studies exploring multiplex-capable SERS-BFNP approaches *in vivo*—all of which are in the cancer field—utilized adoptive transfer of hundreds of thousands to millions of tumor cells into immunocompromised mice, leading to the generation of large palpable tumors 19,21. In comparison, the ^HA^NSG mice in this study possessed at least an order of magnitude fewer endothelial cells, the primary expressers of adhesion molecules in the transplanted adipose tissue. Despite the small population of endothelial cells, simultaneous detection of human ICAM-1 and P-selectin in ^HA^NSG mice *in vivo* using SERS was achieved. Confidence in the accuracy of this result was obtained by subsequent *ex vivo* immunofluorescence staining confirming the expression of these adhesion molecules and the absence of VCAM-1.

Like all nanoparticle-based approaches, there are many challenges ahead in respect to translating research-grade nanoparticle formulations to those suitable for use in humans 52. To achieve this goal, BFNPs must be: synthesized under sterile conditions in GLP facilities to clinical-grade quality, preferably using substances with historical use in humans, such as gold and PEG; assessed for potential immunogenicity; studied in respect to biodistribution, toxicology and pharmacokinetics; standardized to ensure minimal batch-to-batch variation; and rigorously tested for long term potency and stability. However, with research in this area expanding alongside use of antibodies as therapeutics and targeting agents, it is likely that these challenges will be overcome in the near future.

## Conclusions

The demonstrations that SERS-BFNP approaches are fruitful *in vivo*, in combination with the numerous technical advances currently being made, supports SERS as a technology worthy of intense investigation for both invasive and non-invasive molecular imaging of vascular inflammation and atherosclerosis. This study not only opens new avenues for unprecedented multiplexed molecular imaging of vascular inflammation in experimental models, but also highlights the possibility to develop SERS further for clinical use. SERS is one of the few current technologies offering true multiplexing capability for molecular imaging of vascular inflammation, and the development of SERS-based approaches in conjunction with conventional anatomical imaging modalities may facilitate early diagnosis of vulnerable plaques and patient stratification for treatment with anti-inflammatory therapies. The theoretical potential for simultaneous *in situ* imaging of tens of inflammatory biomarkers would also facilitate investigation of new therapeutic avenues by allowing real-time monitoring of inflammatory pathways in patients and advancing the management of many pathologies including cancer, autoimmunity and infectious diseases.

## Supplementary Material

Supplementary figures and tables.

## Abbreviations

BFNP: biofunctional nanoprobes
CVD: cardiovascular disease
CT: computed tomography
CAEC: coronary artery endothelial cells
DCLS: direct classical least squares
AuNP: gold colloid nanoparticles
GJNH: Golden Jubilee National Hospital
GG&C: Greater Glasgow and Clyde
H&E: hematoxylin and eosin
HUVEC: human umbilical vein endothelial cells
IFM: immunofluorescent microscopy
IHC: immunohistochemistry
IVC: individually ventilated cages
ICAM: intercellular adhesion molecule
MRI: magnetic resonance imaging
NPs: nanoparticles
NSG: NOD *SCID* Gamma mice
ORO: Oil Red O
OPO: optical parametric oscillator
PEG: polyethylene glycol
PET: positron emission tomography
SERS: surface-enhanced Raman spectroscopy
TEM: transmission electron microscopy
TNF: tumor necrosis factor
US: ultrasound
VCAM: vascular cell adhesion molecule.
