# Supplementary material for: In vivo multiplex molecular imaging of vascular inflammation using surface-enhanced Raman spectroscopy
Source: Theranostics. 2018 Nov 29;8(22):6195–209. doi: 10.7150/thno.28665 (PMC6299693; doi:10.7150/thno.28665)
Supplement: Supplementary file 1 — Supplementary figures and tables. [file thnov08p6195s1.pdf]

Supplemental Material

***In vivo* multiplex molecular imaging of vascular inflammation using surface-enhanced Raman spectroscopy**

Jonathan Noonan[1,*], Steven M. Asiala[2,*], Gianluca Grassia[1], Neil MacRitchie[1], Kirsten Gracie[2], Jake Carson[3], Matthew Moores[3], Mark Girolami[4,5], Angela C. Bradshaw[6], Tomasz J. Guzik[6,7], Gavin R. Meehan[1], Hannah E. Scales[1], James M. Brewer[1], Iain B. McInnes[1], Naveed Sattar[6], Karen Faulds[2], Paul Garside[1], Duncan Graham[2], Pasquale Maffia[1,6,8]

Supplemental Figures

A

Reporter	Min	Look up table settings	Max
DP	0.35		0.65
BPE	0.35		0.75
PPY	0.35		0.75
PYOT	0.40		0.75

B

Reporter	Min	Look up table settings	Max
BPE	0.35		0.40
PPY	0.35		0.40
PYOT	0.40		0.45

Figure S1. Look up table settings for Raman mapping. (**A**) The minimum and maximum look up table thresholds are shown for *in vitro* / *ex vivo* experiments and (**B**) also for the analysis of adipose grafts isolated following *in vivo* experiments. The minimum threshold was set to exclude any poorly correlating or noisy spectra, whilst the maximum threshold defined the spectral range of false coloring.

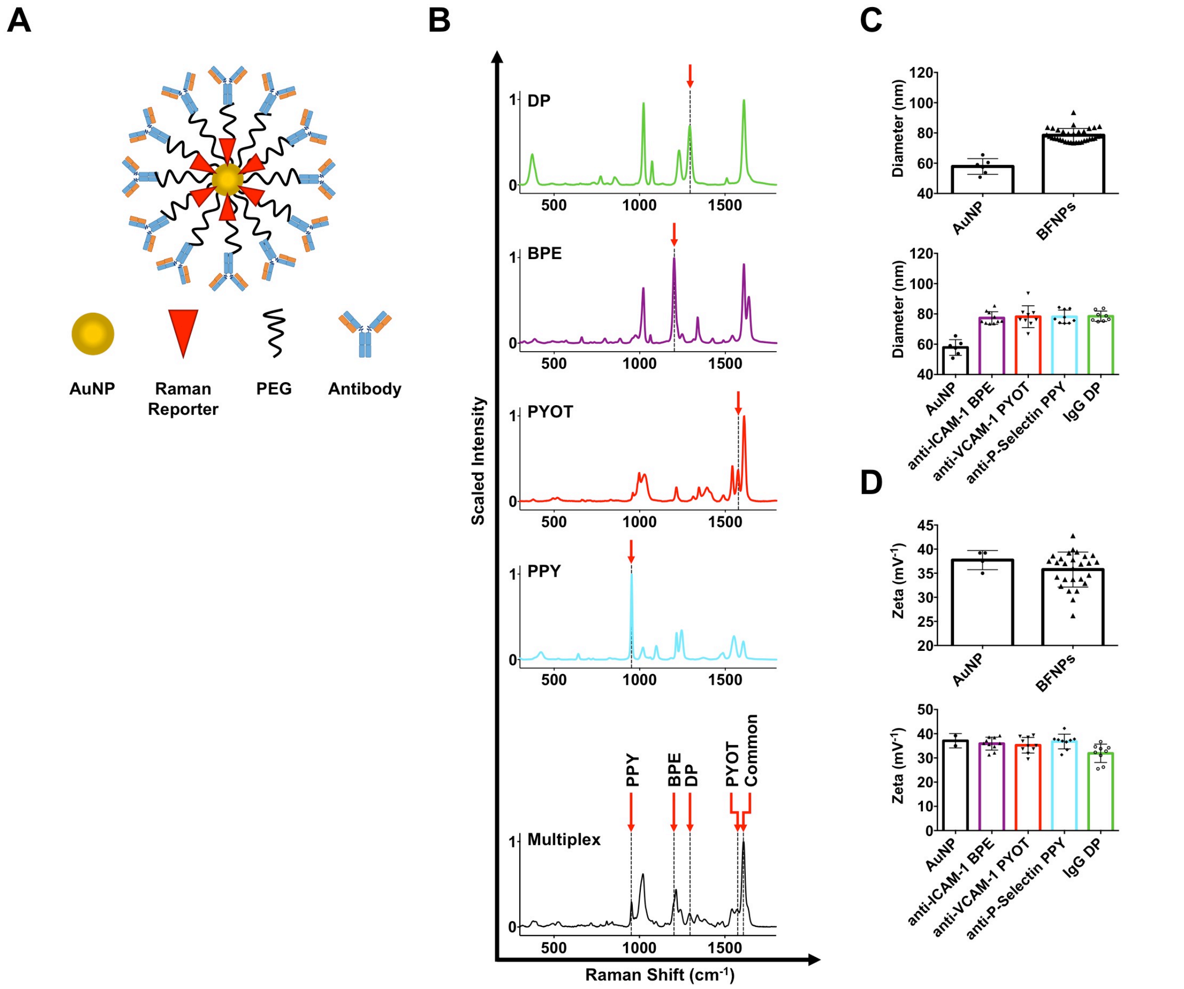

Figure S2. Design and Characterization of biofunctional nanoprobes (BFNP). (**A**) BFNP composition is demonstrated as a schematic depicting a solid gold nanoparticle core (AuNP) conjugated to Raman reporter molecules; this Raman reporter covered core was then conjugated with antibody-functionalized polyethylene glycol (PEG). (**B**) A Raman spectrum from each BFNP conjugated with DP, BPE, PYOT and PPY is shown individually, and in multiplex. The most distinct, unique peaks are highlighted with a red arrow and dotted line for each reporter molecule; the specific peaks used were: DP, 1296 cm^{-1}; BPE, 1202 cm^{-1}, PYOT, 1575 cm^{-1}; PPY, 952 cm^{-1}. In addition, a peak common to all reporter molecules at 1605 cm^{-1} is also highlighted in the Multiplex graph. (**C**) Diameters and (**D**) Zeta potentials of bare gold nanoparticles (AuNP) and fully functionalized BFNP are displayed summarized for all BFNPs (upper panels) and for each BFNP configuration: isotype IgG, DP; anti-ICAM-1, BPE; anti-VCAM-1, PYOT; anti-P-selectin, PPY (lower panels). Values shown are mean +/- SD, with each point representing a single BFNP or AuNP batch.

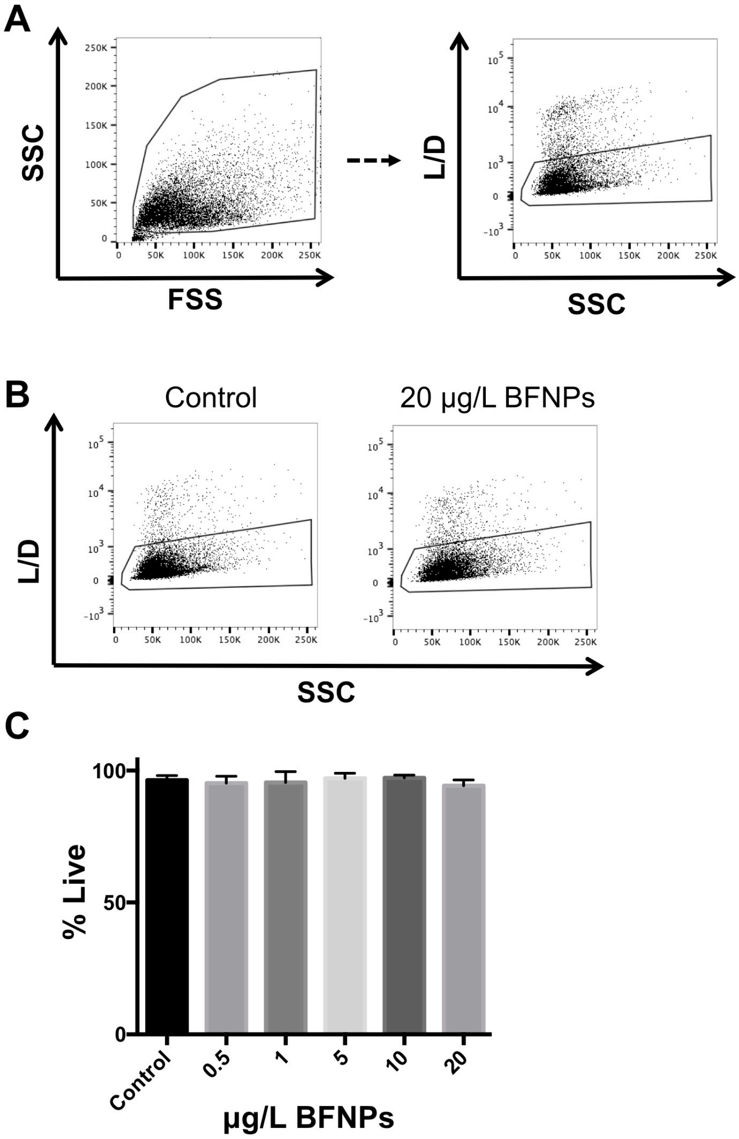

Figure S3. BFNP do not impact on cell viability at the concentrations used in this study. HUVEC were incubated with equal concentration mixtures of isotype-DP, anti-ICAM-1-BPE, anti-VCAM-1-PYOT, and anti-P-selectin-PPY BFNP at total concentrations of 0.5, 1, 5, 10 and 20 µg/L for 24 hours. HUVEC were then washed and stained using a fixable viability Raman reporter and analyzed using flow cytometry. (**A**) Debris was excluded based on forward and side scatter, with live cells defined as dye negative cells. (**B**) Representative plots for control cells cultured in the absence of BFNP and 20 µg/L BFNP condition are shown. (**C**) The percentage of live cells per condition was quantified. Values are mean +/- SD of 3 independent experiments.

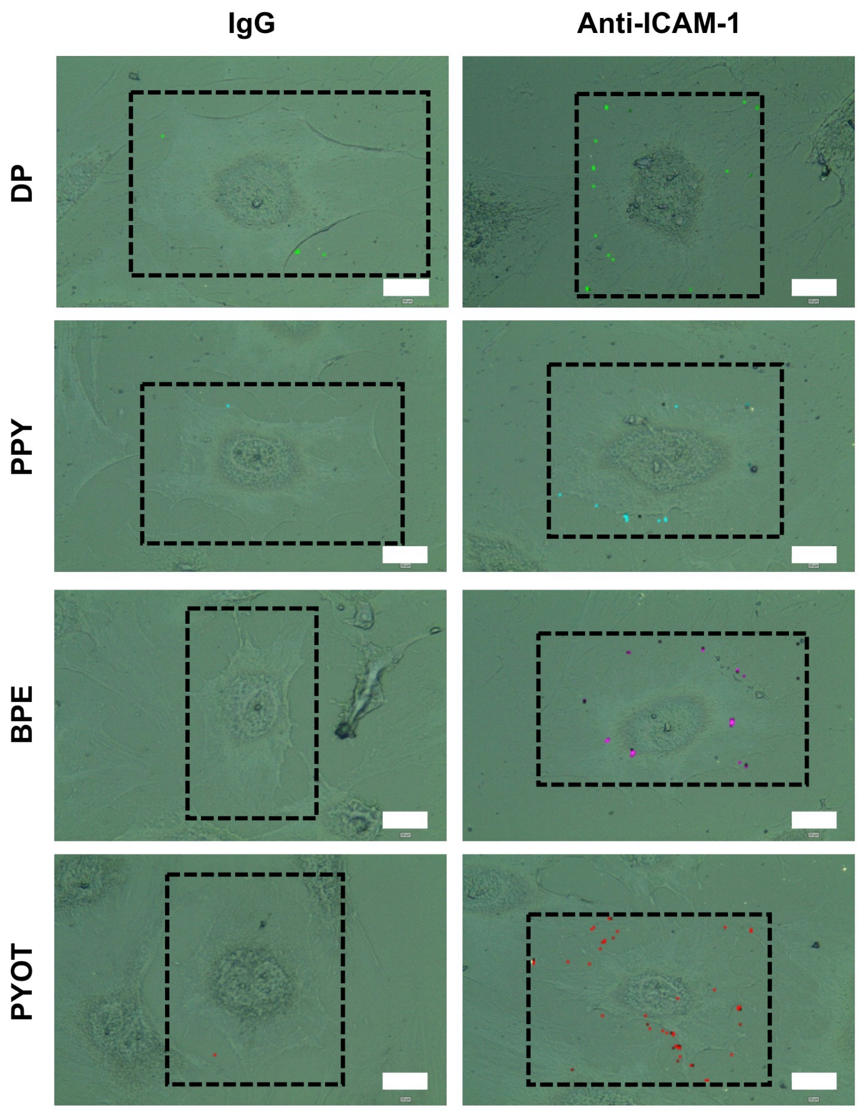

Figure S4. Raman reporter selection does not impact on BFNP functionality. HUVEC were stimulated with 10 ng/ml TNF-α for 24 hours, fixed in acetone, and incubated with isotype control or anti-ICAM-1 BFNP functionalized with the Raman reporter DP, PPY, BPE, or PYOT. Cells were then subject to SERS mapping for isotype or anti-ICAM-1 -DP (green), -PPY (cyan), -BPE (purple) and -PYOT (red) BFNP. The scan areas are indicated by a black box. Results are representative of a single experiment. Optical images are darkfield images. Scale bars = 20 μm.

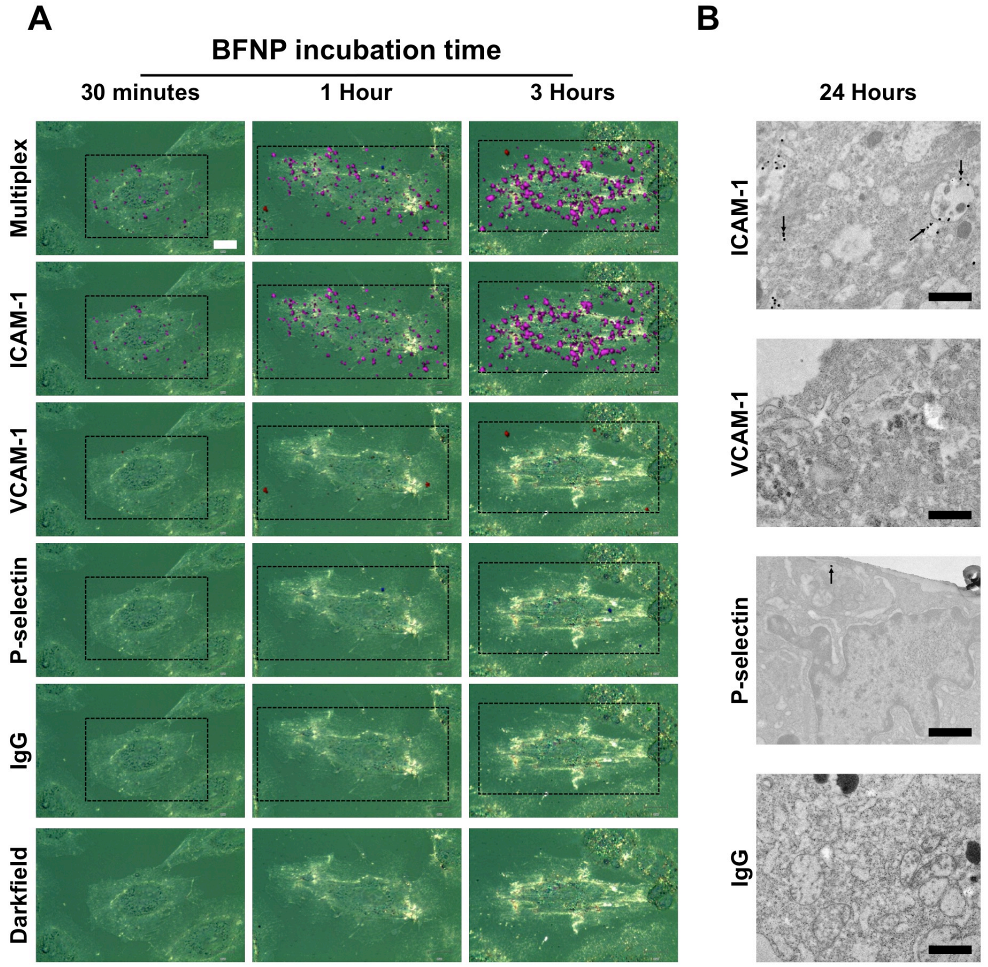

Figure S5. Anti-ICAM-1 BFNP are selectively internalized by CAEC. (**A**) CAEC were stimulated with 10 ng/ml TNF-α for 24 hours and incubated with a mixture of anti-ICAM-1, anti-VCAM-1, anti-P-selectin, and isotype BFNP for 30 minutes, 1 hour or 3 hours. Cells were then washed, briefly fixed in acetone, and subject to SERS mapping for anti-ICAM-1 (purple), anti-VCAM-1 (red), anti-P-selectin (blue) and isotype (green) BFNP. Representative multiplex scans are displayed alongside the individual BFNP channels within these scans (scan area represented by black boxes). Optical images are darkfield images. Scale bars = 20 μm. (**B**) For Transmission Electron Microscopy (TEM), CAEC were stimulated with 10 ng/ml TNF-α for 24 hours and incubated with either anti-ICAM-1, anti-VCAM-1, anti-P-selectin or isotype control BFNP. Cells were washed and then fixed in a glutaraldehyde/paraformaldehyde solution prior to TEM analysis. BFNP appear as uniform black dots, some examples are highlighted with black arrows. Scale bars = 1 μm. SERS microscopy representative of 3 independent experiments; TEM representative of a single experiment.

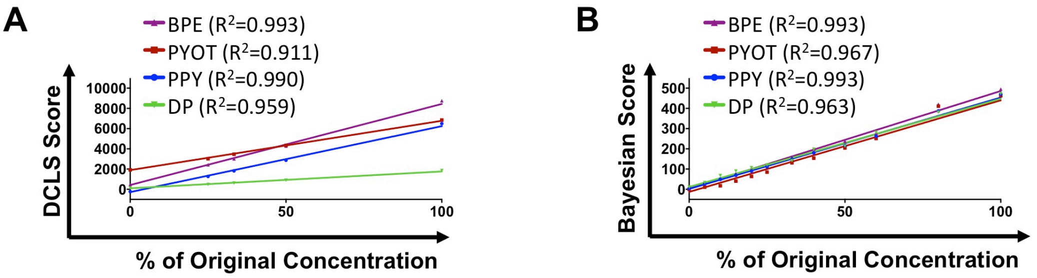

Figure S6. DCLS and Bayesian quantification scores increase with BFNP concentration for mixtures of particles. (**A**) SERS spectra obtained from increasing concentrations of BPE, PYOT, PPY and DP reporter functionalized gold nanoparticles were analyzed using DCLS and (**B**) Bayesian SERS quantification methodologies. Their respective quantitative scores in respect to relative concentration in aqueous mixtures were plotted, and linear regression analysis performed. The R^2 values for each reporter functionalized nanoparticle configuration are shown.

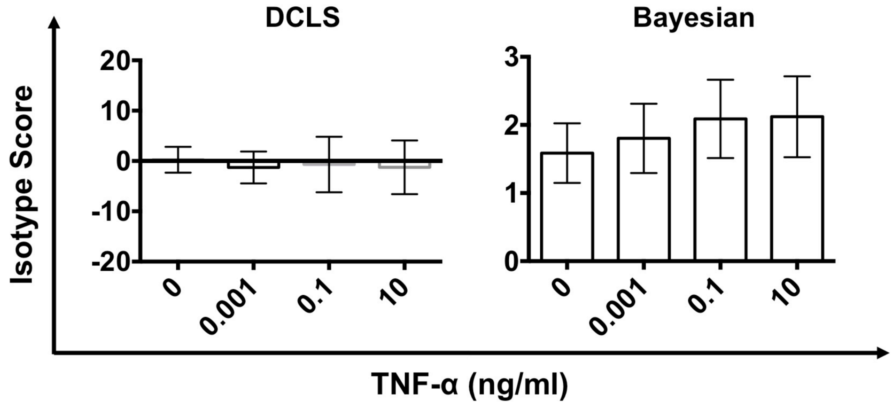

Figure S7. Quantification of isotype BFNP produces near zero DCLS and Bayesian scores. Human umbilical vein endothelial cells were cultured for 6 hours in unstimulated, 0.001, 0.1 or 10 ng/ml TNF-α stimulated conditions, fixed in acetone, and incubated then with isotype control, anti-ICAM-1, anti-VCAM-1, and anti-P-selectin BFNP simultaneously. Cells were then subject to SERS mapping, with each SERS microscopy image spectrally averaged to provide one spectrum per image. DCLS and Bayesian SERS quantification scores were calculated for isotype control BFNP binding in all TNF-α stimulated conditions. Values are mean +/- SD from a single experiment, representative of 3 independent experiments.

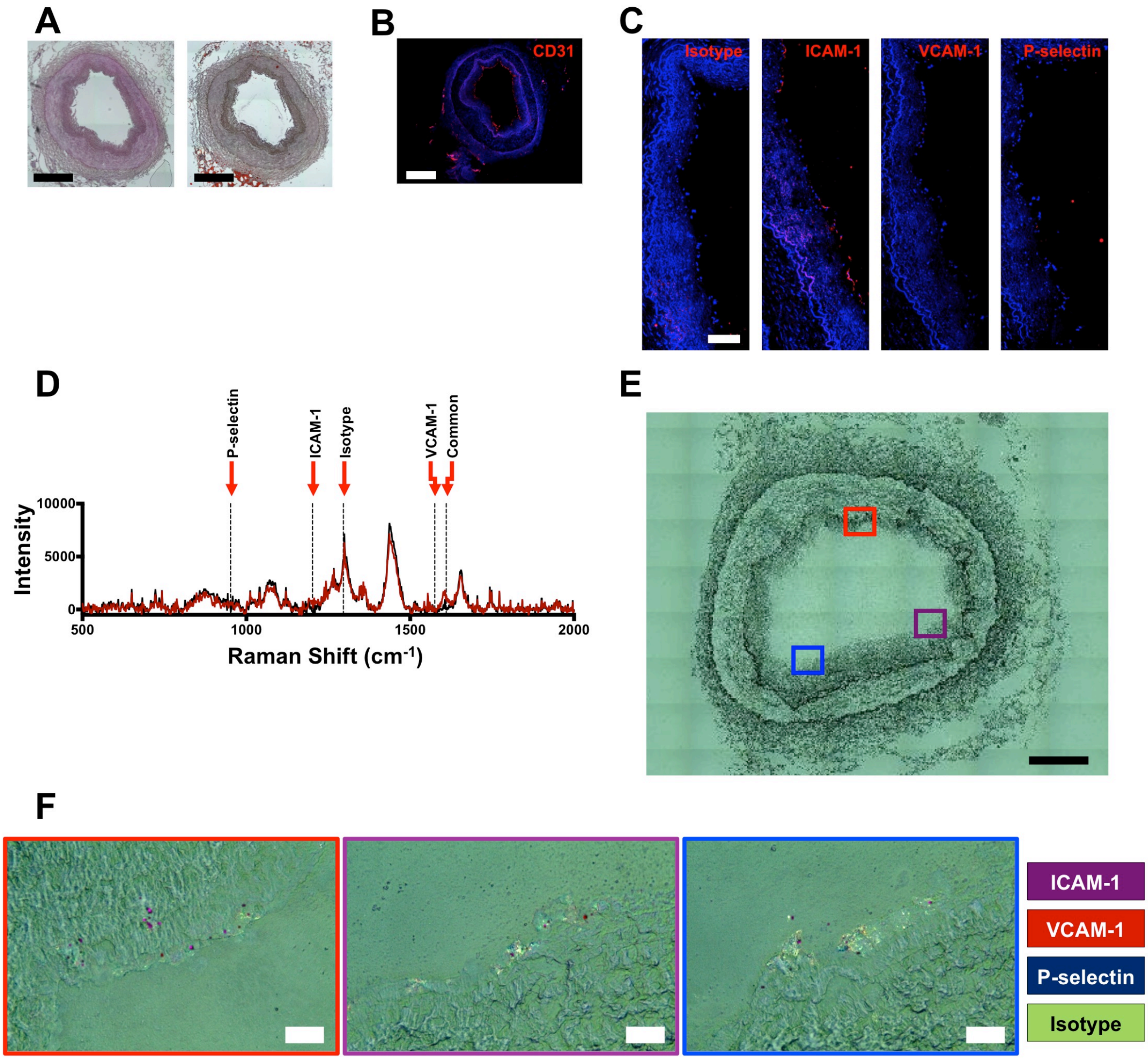

Figure S8. SERS-BFNP imaging of a non-atherosclerotic artery. A human coronary artery was isolated from a patient undergoing heart transplantation surgery. The lumen of the artery segment was then injected with a mixture of anti-ICAM-1, anti-VCAM-1, anti-P-selectin, and isotype control BFNP, sutured closed, and incubated at 37 ^{o}C 5% CO_2 for 12 hours. Sutures were then removed and the artery segment thoroughly washed prior to SERS spectroscopy and subsequent analysis of morphology, expression of adhesion molecules and SERS mapping. Following SERS spectroscopic analysis H&E (**A**; left panel) and oil red O (ORO) staining (**A**; right panel) was carried out to investigate vessel morphology and lipid deposits respectively. (**B**) Immunofluorescent staining for CD31, and (**C**) expression of ICAM-1, VCAM-1 and P-selectin is shown in red. Nuclei were counterstained using Hoechst 33342 (blue). (**D**) SERS spectroscopy was conducted on regions of the artery unexposed (black line) or exposed (red line) to BFNP**.** The unique peaks and common peak used to identify each BFNP configuration are highlighted with a red arrow and dotted line and

labeled accordingly. Spectroscopic results are displayed as spectra averaged from at least 3 different locations within the tissue segments. (**E**) A representative darkfield image is shown of a section of the artery and regions subject to SERS mapping for anti-ICAM-1 (purple), anti-VCAM-1 (red), anti-P-selectin (blue), and isotype (green) BFNP are highlighted with red, blue and purple boxes corresponding to SERS maps in **F**. Results shown are from a single coronary artery from a patient undergoing heart transplantation. Optical images in **E** & **F** are darkfield images. Scale bars: **A** & **B** = 500 μm; **C** = 100 μm; **E** = 500 μm; **F** = 20 μm.

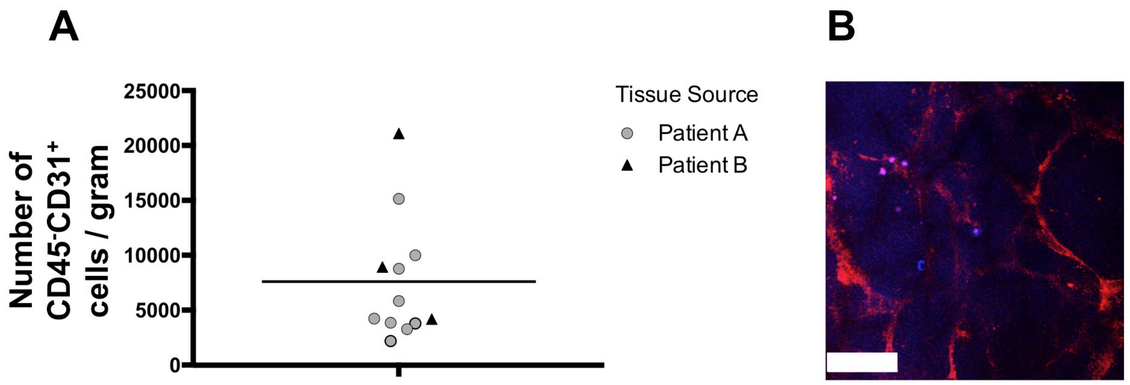

Figure S9. Adipose grafts from ^{HA}NSG mice contain viable and perfused human blood vessels. (**A**) Implanted human subcutaneous fat was excised and analyzed by flow cytometry to confirm the presence of human endothelial cells. Cell numbers were normalized to the weight of isolated grafts and are expressed per gram. Approximately 7500 human CD45-CD31+ (endothelial) cells were present in adipose implants, indicating the presence of human vasculature. The data shown is from multiple implants generated using tissue provided by two different tissue donors. (**B**) Engrafted mice were injected intravenously with fluorescent anti-human CD31 antibody (red) and imaged using a multiphoton microscope, second harmonic generation can also be observed (blue). Labeling of blood vessels *in situ* confirmed implants were perfused as a result of successful anastomoses with the host circulatory system. Scale bar = 100 μm.

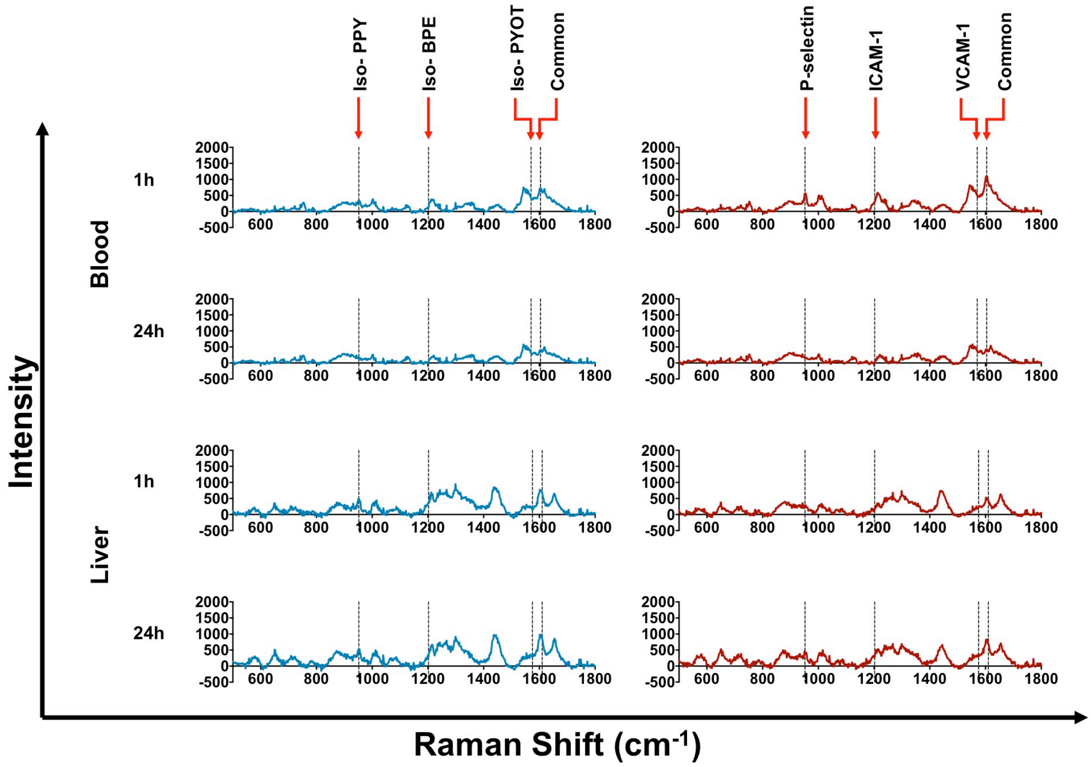

Figure S10. Distribution of BFNP in the blood and liver following intravenous injection. Following engraftment of human adipose tissue, ^{HA}NSG mice were allowed to recover for 3 weeks. Mice were then injected intravenously with 5 µg of human recombinant TNF-α 4 hours prior to receiving an intravenous injection of BFNP. SERS spectra were acquired from blood samples and livers of anaesthetized ^{HA}NSG mice receiving a mixture of isotype-PPY, -BPE and -PYOT (blue spectra), or anti-P-selectin-PPY, anti-ICAM-1-BPE, anti-VCAM-1-PYOT BFNP (red spectra) 1 and 24 hours post BFNP injection.

Supplemental Table

Patient	Reason for transplant	Relevant medical history	Age	Sex	Observations	Results of SERS-BFNP imaging
1	Chronic heart failure	Hypertensive, previous myocardial infarction	48	Female	Artery contained an atherosclerotic plaque	Detection of ICAM-1, VCAM-1 and P-selectin.
2	Hypertrophic cardiomyopathy	Previously received percutaneous coronary intervention to left anterior descending coronary artery	56	Male	Artery contained a calcified atherosclerotic plaque	Detection of P-Selectin.
3	Biventricular Dilation, suspected Dilated Cardiomyopathy	-	50	Female	None	No specific detection of adhesion molecules, but presence of BFNP common peak suggestive of low level BFNP binding.
4	Dilated Cardiomyopathy	-	55	Male	None	No BFNP signals observed

Table S1. Clinical characteristics of human coronary arteries and summarized SERS-BFNP imaging results.
